# Proton Pump Inhibitors and Clostridium Difficile Infection: Are We Propagating an Already Rapidly Growing Healthcare Problem?

**DOI:** 10.4021/gr575w

**Published:** 2013-10-31

**Authors:** Rashmee Patil, LeAnn Blankenship

**Affiliations:** a Department of Internal Medicine, Lutheran HealthCare, 150 55th Street, Brooklyn, NY, USA

**Keywords:** Proton pump inhibitors, *Clostridium difficile* infection, Recurrence, Cause-effect relationship, Treatment, Complications, Management

## Abstract

Proton pump inhibitors (PPIs) have been associated with *Clostridium difficile* infection (CDI) in several recent studies. The exact mechanism through which PPIs may cause *Clostridium difficile* infection is not well understood. One potential mechanism to explain this association may be that elevated gastric pH levels facilitate the growth of potentially pathogenic upper and lower gastrointestinal tract flora. Although *Clostridium difficile* spores are acid resistant, vegetative forms are susceptible to acidity. Higher gastric PH therefore increases vegetative bacteria counts in the small and large intestine. Other potential mechanisms include impairment of leukocytes and other immune responses and antimicrobial properties of PPIs. In recent years, much research has been contributed to prove the relationship between PPIs and CDI as causal. Most studies however, fail to prove causality due to the use of antibiotics and other medications during time of initial diagnosis of CDI. PPIs continue to also be one of the most heavily prescribed drugs in our country. As primary and recurrent infection caused by *Clostridium difficile* continues to rise, more data must be collected to determine better treatment, overall management, and the role that PPIs may play in its propagation.

## Introduction

Proton pump inhibitors (PPIs) are one of the most prescribed groups of drugs globally [1]. PPIs are effective for the treatment of all acid-related disorders. They are also indicated in ICU patients with coagulopathy, patients on mechanical ventilation, and patients with a history of peptic ulcer disease (particularly those on NSAIDs or antiplatelet therapy) [1, 2]. The use of PPIs has increased dramatically despite concerns that PPIs are overprescribed both in primary care and in hospitals. Moreover, concerns have been raised about the potential long-term effects of these drugs. PPIs have been associated with significant interactions with other drugs and complications such as fractures, interstitial nephritis, pneumonia, and enteric infections, namely *Clostridium difficile* infection (CDI) [3-5]. CDI has recently emerged as a major public health problem with current estimates suggesting a point prevalence of 13.1/1,000 in the inpatient population [5]. Studies have reported increases in both incidence and mortality of CDI. The increase in incidence of CDI has been attributed to an ageing population, increase in use of antibiotics and acid suppressive drugs. PPIs are postulated to increase the proliferation of spores and change the acidic environment of the stomach which permits spores to survive intraluminally [6-8]. The role of gastric acid suppression therapy has gained much interest recently as a risk factor for CDI. Many recently published meta-analyses have suggested an association between gastric acid suppression therapy with PPIs and CDI [9-11].

## PPI Overuse

PPIs are highly effective acid suppressive drugs but in recent years have become widely overprescribed [12]. Gastric acid inhibits the germination of ingested spores and the survival of *C. difficile*, and recent studies have elucidated that gastric acid suppressive agents, such as PPIs, increase the risk of CDAD development in hospitalized patients [13]. The mechanism by which PPI may increase the risk of CDI is not well understood. It has been suggested that reduced acidity of the gastric contents resulting from treatment with acid-suppressive therapy allows vegetative bacteria to pass into the small intestine [14]. Although there is some evidence to support this hypothesis, it has also been demonstrated that *C. difficile* spores are unaffected by normal gastric pH, and the relative contributions of spores and vegetative bacteria to the pathogenesis of disease are currently unclear. PPIs have also been shown to inhibit the phagocytic neutrophil response to *Escherichia coli*, leading to speculation that altered host immune functions may increase the risk of incidence and recurrence of CDI [15-17]. Alternatively, PPI metabolites may exhibit antibacterial effects in the GI tract. The inhibitory properties of sulfide analogues formed after proton-dependent activation of PPI against *Helicobacter* spp. have been documented, but the susceptibility of bacterial strains common to the normal flora was not evaluated, and the specific mechanism of bactericidal activity is unknown. Further research is needed to clarify whether PPI has sufficient activity against the normal flora to have a meaningful impact on the risk of CDI [18-20].

## Relationship Between PPI Use and CDI

*Clostridium difficile* is a Gram-positive, anerobic, spore-forming bacillus that is the most common infectious cause of healthcare-associated diarrhea in developed countries. The recent emergence of an epidemic strain, termed North American pulsed-field gel electrophoresis type 1, or NAP1 has been associated with large outbreaks of CDI in North America and Europe [21]. In addition to traditional risk factors, such as exposure to antibiotics and increased underlying disease severity, several recent studies have reported an association between PPIs and nosocomial or community-associated CDI. Because PPIs are often used in the absence of clear indications, it might be feasible to reduce the use of these agents as a control strategy for *C. difficile*. However, the role of PPIs in the pathogenesis of CDI is controversial because some studies have not associated PPIs with *C. difficile* and the mechanisms by which acid-suppressive medications might promote CDI are unclear [22].

Current research regarding the causal relationship between PPIs and CDI has varied from paper to paper. In the July 2012 issue of the *American Journal of Gastroenterology*, two large meta-analyses regarding this relationship were published. In Janarthanan et al, there was a reported 1.69 times higher risk of CDI with PPI use. Results were derived from a total of 288,620 patients from 23 studies. In a similar meta-analysis, Kwok et al found an increased odds of acquiring CDI by 1.74 in a pool of 313,000 patients. Using slightly different methodologies, both studies elucidated an increased risk of CDI in PPI-using patients vs. those not using PPIs [23, 24].

Though the clear causal link between CDI and PPI use has not been established, recent studies have identified a link between PPI use and recurrent *C. difficile* in a patient population. In a study by Linsky et al, there was a 42% increased risk of recurrent CDI related to PPI use. Given the morbidity and cost associated with recurrent CDI and the lack of readily modifiable risk factors, the findings have important clinical implications. The data presented support the need for critical assessment of PPI use in patients being treated for CDI as well as further research to test this association [1, 5, 9].

In a similar study by Jin et al, older age (> 65 years) and a low serum albumin level (< 2.5 g/dL) were identified as risk factors for CDI recurrence. The concomitant use of PPIs further enhanced the risk of recurrence. Of these risk factors, the use of PPIs is modifiable, and thus, it is appropriate to review constantly the necessity for concomitant use of PPIs in patients with CDI.

As with differentiation between recurrent CDI, there is also a need for more prospective studies comparing community-associated CDI with healthcare-associated infection. Community-acquired infection is an important health care problem with a distinct epidemiology in comparison to healthcare facility-associated CDI [9, 24].

In addition to antimicrobial exposure, other previously unrecognized factors appear to have a role in CDI pathogenesis, including remote health care exposures, community transmission in the home, and medications with anti-inflammatory properties. A complete assessment for these factors may be helpful when determining the risk of CDI in a community-based patient with diarrhea who does not have the typical risk factors of recent hospitalization or antimicrobial exposure. Studies have also shown that PPIs may have more impact on community-associated CDI than in hospital-acquired; however, the effects are almost interchangeable given the widely used nature of the drug, though PPIs may effect community strains more than hospital-acquired ones [15, 22, 23].

## Conclusion

PPI use has been linked to an increase in CDI over the past few years. Though the exact mechanism is unclear and there has yet to be a study to prove causality, the overuse of PPIs in inpatient and outpatient settings can explain the relationship. Future studies need to focus on evaluating the dosage and duration of PPI use and measuring its effects on CDI independent of other variables, including antibiotic use, in order to show strength in the association. CDI remains a large healthcare burden and as new strains begin to proliferate and resistance becomes an added factor, we must determine the factors that we can control to ensure safer medical practices.
